# Loss of the SxxSS Motif in a Human T-Cell Factor-4 Isoform Confers Hypoxia Resistance to Liver Cancer: An Oncogenic Switch in Wnt Signaling

**DOI:** 10.1371/journal.pone.0039981

**Published:** 2012-06-29

**Authors:** Hironori Koga, Orkhontuya Tsedensodnom, Yoshito Tomimaru, Evan J. Walker, Han Chu Lee, Kang Mo Kim, Hirohisa Yano, Jack R. Wands, Miran Kim

**Affiliations:** 1 Liver Research Center, Rhode Island Hospital and the Warren Alpert Medical School of Brown University, Providence, Rhode Island, United States of America; 2 Department of Internal Medicine, Asan Medical Center, University of Ulsan College of Medicine, Seoul, Korea; 3 Department of Pathology, Kurume University, Kurume, Japan; Ohio State University Comprehensive Cancer Cente, United States of America

## Abstract

**Purpose:**

Aberrantly activated Wnt/β-catenin signaling is important in hepatocellular carcinoma (HCC) development. Downstream gene expressions involving the Wnt/β-catenin cascade occur through T-cell factor (TCF) proteins. Here, we show the oncogenic potential of human TCF-4 isoforms based on the expression of a single conserved SxxSS motif.

**Methods:**

We investigated the TCF-4J and K isoform pair characterized by the presence (K) or absence (J) of the SxxSS motif. The mRNA expression profiles were examined in 47 pairs of human HCCs and adjacent non-cancerous liver tissues by RT-PCR. Proliferation, sphere assays and immunoblot analysis were performed under normoxia and hypoxia conditions. The ability of HCC cells overexpressing TCF-4J (J cells) and K (K cells) to grow as solid tumors in nude mice was explored.

**Results:**

TCF-4J expression was significantly upregulated in HCC tumors compared to corresponding peritumor and normal liver and was preferentially expressed in poorly differentiated HCCs. In contrast, TCF-4K was downregulated in those same HCC tumors. TCF-4J-overexpressing HCC cells (J cells) revealed a survival advantage under hypoxic conditions, high proliferation rate and formation of aggregates/spheres compared to overexpression of TCF-4K (K cells). The hypoxic J cells had high expression levels of HIF-2α and EGFR as possible mechanisms to promote tumorigenesis. Increased stability of HIF-2α under hypoxia in J cells was associated with a decreased level of von Hippel-Lindau (VHL) protein, a known E3 ligase for HIF-αs. In a xenograft model, the J cells rapidly developed tumors compared to K cells. Tumor tissues derived from J cells exhibited high expression levels of HIF-2α and EGFR compared to the slow developing and small K cell derived tumors.

**Conclusions:**

Our results suggest that the specific TCF-4J isoform, which lacks a regulatory SxxSS motif, has robust tumor-initiating potential under hypoxic conditions.

## Introduction

The Wnt/β-catenin signaling pathway plays a crucial role in cell fate determination and stem cell renewal in adult tissue [1], [2]. Genetic and/or epigenetic deregulation of this pathway leads to aberrant nuclear accumulation of β-catenin where it binds with T-cell factor-4 (TCF-4) to form a transcriptional complex [3]; this event drives gene expression such as c*-Myc*, *cyclin D1*, and *EPCAM* [4], [5], [6] that contributes to a malignant phenotype.

Hepatocellular carcinoma (HCC) is the third common cause of cancer mortality worldwide [7]. Aberrantly activated Wnt/β-catenin signaling due to overexpression of upstream components of this pathway such as Frizzled (FZD) receptors and Wnt ligands is a common early event in the molecular pathogenesis of this disease [8], [9], [10]. However, the involvement of the canonical Wnt TCF-4 effector proteins in this process has yet to be examined.

The human TCF-4 gene (*TCF7L2*) consists of 17 exons and has several alternative splicing sites in exons 13–17 and exon 4 [11]. The alternative splicing sites in the central domain of TCF-4 can also generate isoforms with or without the conserved LVPQ and SxxSS motifs located at the end of exon 7 and the beginning of exon 9, respectively [11], [12]. Recently, we identified and characterized 14 (12 of which are unique) TCF-4 isoforms derived from human HCC cell lines [13]. Among such isoforms, three structurally identical pairs (TCF-4J and K; TCF-4A and B; TCF-4G and H) were observed that differed only by the presence (K, A, and H) or absence (J, B, and G) of a SxxSS motif. In this context, two pairs of isoforms (TCF-4A and B, and TCF-4H and G) are short forms, whereas TCF-4K and J are long forms due to the inclusion of a C-terminal tail (exon 13–17) [13]. Previous studies suggest that the SxxSS motif may modulate transcriptional activity of TCF-4 [12]; however, both the cell-based and *in vivo* functional consequences of the SxxSS motif expression as well as subsequent gene regulatory activity have not been determined.

Emerging evidence indicates that both embryonic (ES), adult and inducible pluripotent stem (iPS) cells [14] prefer hypoxic conditions for growth and survival [15]. Hypoxia generates diverse cellular signals through the stabilization of hypoxia-inducible factors (HIF-αs) such as HIF-1α and HIF-2α. Recent studies reveal that HIF-αs interact with β-catenin and, therefore, may regulate TCF-4-driven gene expression not only in stem/progenitors but also tumor cells experiencing hypoxic conditions during rapid growth [16], [17]. These findings imply that Wnt/β-catenin/TCF-4 signaling may be directly regulated by the professional oxygen-sensing system in the nucleus. For instance, stabilized HIF-2α, a partner of β-catenin and often found in the hypoxic core of the tumor, upregulates the expression of epidermal growth factor receptor (EGFR) and may contribute to tumor growth [18]. In human HCC, the HIF-αs are involved in the multi-step process of tumor dedifferentiation via promotion of angiogenesis [19]. Accordingly, we determined if different functional properties of TCF-4 isoforms associated with the HCC malignant phenotype were regulated in the context of a SxxSS motif-dependent mechanisms under conditions of oxygen deprivation.

## Materials and Methods

### Ethics Statement

Full ethical approval was obtained for all human sample collections from either the Asan Medical Center Ethics Committee or the Kurume University Ethics Committee. All samples were obtained with written consent. All animal experiments were conducted in accordance with the NIH Guidelines for the Care and Use of Laboratory Animals and were approved by the Lifespan Animal Welfare Committee of Rhode Island Hospital, Providence, RI (permit number A3922-01).

### Detection of TCF-4 Isoforms in HCC Tumors by RT-PCR

Preparations of human TCF-4A, B, J and K-myc plasmids have been previously described [13]. Two independent RT-PCR analyses were performed using 47 pairs of human HCCs (Tables 1 and 2) as previously described [13].

### Cell Lines and Cultures

Human cell lines Hep3B, Huh7, HepG2, and HEK293 were obtained from the American Type Culture Collection (ATCC, Manassas, VA). FOCUS cell line was obtained from Dr. Wands (Liver Research Center, Rhode Island Hospital, RI). HAK-1A and HAK-1B cells were provided by Dr. Yano (Kurume University School of Medicine, Japan). The immortalized hepatocyte derived cell line OUMS-29 was a kind gift from Drs. Namba and Kobayashi (Okayama University, Japan). Hep3B, Huh7, HepG2, and FOCUS cell lines are hepatitis B virus (HBV)-related HCC cells. HAK-1A and HAK-1B cells are hepatitis C virus (HCV)-related HCC cell lines. All cells have been described and employed in previous studies (Hep3B [20], Huh7 [21], HepG2 [20], HEK293 [22], FOCUS [23], and HAK-1A and HAK-1B [24]). To generate stable transfectants, HAK-1A cells were transfected with the TCF-4 or empty vector plasmid using TransIT-LT1 Reagent (Mirus Bio Co., Madison, WI) and selected by G418.

### Hypoxia Induction

Hypoxic conditions (1% oxygen) were produced by an incubator equipped with an oxygen concentration regulator system (ASTEC, Fukuoka, Japan). Alternatively, cells were exposed to hypoxia-mimic chemical cobalt chloride (CoCl_2_, Sigma-Aldrich, St. Louis, MO) [25].

### Confocal Laser Scanning Microscopy

Cells, grown on 35 mm diameter Glass Bottom Dishes (MatTek, Ashland, MA), were fixed with cold acetone/methanol (1∶1) for 10 min, and then washed in PBS containing 0.05% Tween 20 (PBS-T). Nonspecific reactions were blocked with Protein Block Serum-Free (DAKO North America, Carpinteria, CA) and then incubated with a mouse anti-Myc-tag antibody (Santa Cruz Biotechnology, Santa Cruz, CA) or a rabbit anti-HIF-2α antibody (Abcam, Cambridge, UK) at 4°C overnight. After washing in PBS-T, the specimens were treated with Alexa Fluor goat anti-mouse or goat anti-rabbit IgG (H+L) antibody (Molecular Probes, Eugene, OR) for 40 min at RT, and then counterstained by VECTASHIELD Mounting Medium with 4′,6-diamino-2-phenylindole (DAPI) (Burlingame, CA). The Zeiss LSM510 Confocal Laser Scanning Microscope (Carl Zeiss MicroImaging, Inc., Thornwood, NY) equipped with the user interface software Zen 2008 was used to visualize the immunofluorescence staining for Myc-tag or HIF-2α and nuclear localization was provided by DAPI. The negative controls were obtained by incubating cells with non-specific mouse IgG or rabbit IgG as described above.

### Immunoblot Analysis and Immunoprecipitation

The primary antibodies used were against for Myc-tag, β-catenin, actin, γ-tubulin, lamin A/C, HIF-1α, c-myc, and ubiquitin (Santa Cruz Bio, Santa Cruz, CA), TCF-4, EpCAM, and von Hipple-Lindau (VHL) (Cell Signaling Technology, Beverly, MA), HIF-2α (Abcam, Cambridge, UK), and keratin-19 (K-19) (DAKO, Carpinteria, CA). A TCF-4 rabbit derived mAb (Cell Signaling Technology, Cat# 2569) recognizes surrounding the Leu330 of human TCF-4 and detects endogenous TCF-4 proteins including both the short and long isoforms. For immunoprecipitation, cell extracts were prepared as previously described [26]. Total cell lysate or nuclear extract were incubated with antibodies against HIF-1α, HIF-2α, and VHL followed by Recombinant Protein G Agarose (Invitrogen, Carlsbad, CA). Immunoblots were probed with antibodies to ubiquitin, HIF-1α, HIF-2α, and VHL and detected with HRP-labeled anti-mouse or anti-rabbit IgG (Amersham Pharmacia Biotech, Buckinghamshire, UK) using ECL Advanced kit (Amersham). Positive signals were captured by the Image analyzer LAS-1000plus (Fujifilm, Tokyo, Japan), and the band intensity of protein was determined using Image Gauge version 3.45 (Fujifilm).

### Cell Proliferation Assay

Cells were seeded into 24-well plates at a density of 2.5×10^3^/well. A colorimetric assay (CellTiter 96® Aqueous One Solution Cell Proliferation Assay; Promega, Madison, WI) was performed at days 0, 1, 3, 5, and 7, and the signals were measured using Spectra Max M5 (Molecular Devices, Sunnyvale, CA).

### Anchorage-independent Growth (Sphere Assay)

Single-cell suspensions were put into Ultra Low Attachment 6-well plates (Corning, Lowell, MA). After 27 days, the number of cell aggregates/spheres per well was quantified and photographed under the Zeiss Axiovert 200 M Fluorescence Microscope (Carl Zeiss MicroImaging). The area of the cell aggregates was measured using the MetaMorph 6.0 software (Molecular Devices).

### Evaluation of Protein Stability

Cells were exposed to 5 µg/mL cycloheximide (CHX; Sigma-Aldrich) for 0 or 60 min at the last phase of the 48 h CoCl_2_ treatment. Total cell lystes were used for immunoblot analysis to evaluate HIF-1α and HIF-2α expression levels after protein synthesis was inhibited by CHX.

### Xenograft Tumor Model in Nude Mice

HAK-1A-derived stable clones were used; parental HAK-1A cells do not form tumors in nude mice, and are therefore suitable for assessment of malignant transformation following stable transfection with TCF-4 isoforms [24]. Cells (1×10^6^) were sub-cutaneously injected into the back of 5-week old male BALB/c nude mice (n  = 12) (Taconic Farms, Cranbury, NJ). The tumor size was measured twice per week, and the tumor volume (mm^3^) was estimated using the equation length × (width)^2^×0.5. When the longer diameter reached 10 mm, the mice were sacrificed and the tumors were prepared for subsequent analysis.

### immunohistochemistry of Xenograft Tumors

Paraffin-embedded tissue sections were deparaffinized and heated in 10 mM citrate buffer at 120°C for 5 min for antigen retrieval. The sections were pre-blocked by Protein Block Serum-Free (DAKO) and incubated with primary antibodies for HIF-2α (Abcam) and EGFR (D38B1 XP™) (Cell Signaling Technology). After washing, the sections were incubated with EnVision secondary antibodies labeled with HRP-polymer complexes (DAKO) and visualized by 0.1% 3–3′′-diamino-benzidine-tetrahydrochloride. The cell nuclei were counterstained with hematoxylin. Specimen incubated with mouse or rabbit IgG were set as the negative controls.

### Statistical Analysis

Statistical significance was performed by the Mann-Whitney U test using GraphPad Prism 4.0 software (San Diego, CA). *P*<0.05 was considered statistically significant. The Pearson’s correlation coefficient was used to calculate correlation between expression of TCF-4J and K.

## Results

### Differential Expression Profile of TCF-4J and TCF-4K in Human HCC Tissues

**Figure 1:**
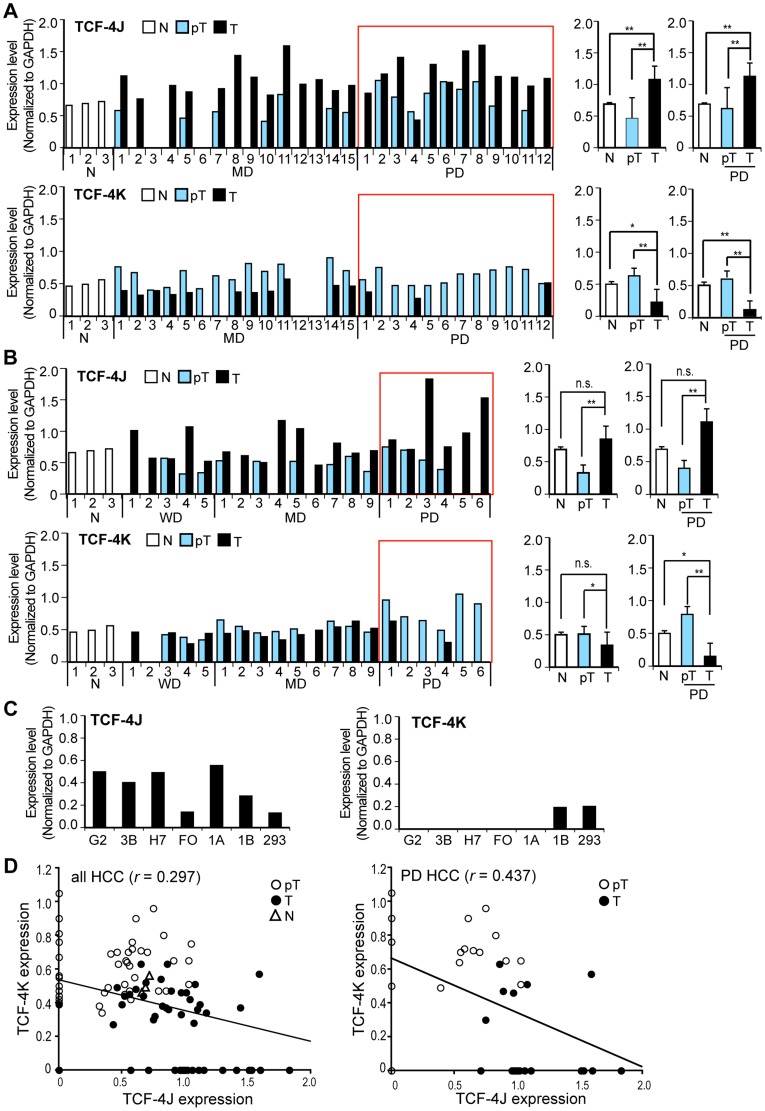
Expression of TCF-4J and TCF-4K mRNAs was different in human HCCs. (A) Comparative analysis of TCF-4J and K mRNA expression levels in 27 HBV-related HCC tumors (T) adjacent peritumor tissue (pT) and histologic normal liver (N) by RT-PCR. Values are normalized to GAPDH. Red rectangles denote poorly differentiated (PD) HCC. WD, well differentiated; MD, moderately differentiated. Statistical results from all tissues or PD HCC are expressed as mean + SD (right panel). (B) Another 20 HCC tumors including five WD HCCs from a different clinical site. Seventeen individuals had HCV-related chronic liver disease, and the remainder had chronic HBV infection. See also Tables 1 and 2. (C) Expression levels of TCF-4J and K mRNA in human cell lines HepG2 (G2), Hep3B (3B), Huh-7 (H7), FOCUS (FO), HAK-1A (1A), HAK-1B (1B), OUMS-29 (OU), and HEK293 (293). (D) Inverse correlation between TCF-4J and K expression in all HCC (left panel) and PD HCC (right panel). Note the weak inverse correlation in all cases (*r*  = 0.297), while increased inverse correlation in PD HCC (*r*  = 0.437). *p<0.01; **p<0.05.

Expression of TCF-4 isoforms with or without the SxxSS motif may influence their transcriptional activity [12], [13], [27]. Therefore, we determined if three pairs of isoforms (TCF-4A and B; TCF-4G and H; and TCF-4J and K) exhibited different expression profiles that may suggest SxxSS-dependent regulation in human HCC tumors. The relative mRNA levels of these TCF-4 isoforms in 27 pairs of HCC tumors and corresponding adjacent liver tissue obtained from chronic HBV-related (26/27) disease were measured. Comparisons were made to three normal liver specimens by semi-quantitative RT-PCR (Fig. 1A, Fig. S1A and C, and Table 1) as previously described [13]. TCF-4J expression was significantly upregulated in HCC tumors compared to peritumor tissue and normal liver. Among the 27-paired samples, 85% had increased TCF-4J expression in HCC compared to matched-peritumor tissue. In contrast, TCF-4K was significantly downregulated in tumors compared to normal liver and peritumor tissue (Fig. 1A). Furthermore, a similar expression profile of these TCF-4 isoforms was observed in a second cohort of HCC-adjacent uninvolved tissue pairs from individuals where 85% (17/20) of the tumors were related to chronic HCV infection (Fig. 1B, Table 2). However, this differential expression pattern was not observed in the two other pairs of short isoforms (TCF-4A and B, G and H) (Fig. S1). TCF-4A, a short form of K was significantly downregulated in HBV-related HCC tumor compared to normal liver as well as peritumor tissue. In addition, the expression level of TCF-4B, a short form of J, was found to be similar among normal, tumor, and peritumor tissues (Fig. S1A). Furthermore, the other pair of TCF-4 isoforms (G and H) also revealed no differences of their expression levels among normal, HCC tumor, and peri-tumor tissues (Fig. S1C). In HCV-related HCC tissues, however, all four isoforms (TCF-4A and B, G and H) were significantly downregulated in both peritumor and tumor tissues compared to normal liver; in addition no difference was found between tumor and peritumor samples (Fig. S1B and D). These results indicated that the influence of SxxSS motif on generation of a HCC malignant phenotype may be associated with the long (TCF-4J and K), not short isoforms (TCF-4A and B, G and H).

**Table 1:** Patient and tumor characteristics in South Korean patients.

No	Age/Sex	Virus	Tumor diagnosis	Max. dia. (cm)	Grade	Vas. Inv.	Peri-T liver
1	46/M	B	HCC/CCC	6.8	MD	(−)	LC
2	56/M	B	HCC	2.7	MD	(−)	CH
3	47/M	B	HCC	3.5	MD	(−)	LC
4	57/M	B	HCC	1.7	MD	(−)	CH
5	55/M	B	HCC	3.0	MD	(−)	LC
6	67/M	B	HCC	8.5	MD	(−)	CH
7	61/M	B	HCC	3.0	MD	(−)	LC
8	37/M	B	HCC	9.5	MD	(−)	CH
9	53/M	B	HCC	4.2	MD	(−)	LC
10	57/M	B	HCC	16.0	MD	(+)	CH
11	42/M	B	HCC	4.7	MD	(−)	LC
12	50/M	B	HCC	4.5	MD	(−)	LC
13	41/M	B	HCC	4.0	MD	(−)	CH
14	44/M	B	HCC	3.8	MD	(−)	LC
15	69/F	B	HCC	8.0	MD	(−)	LC
16	45/M	B	HCC	15.0	PD	(−)	LC
17	55/M	B	HCC	11.0	PD	(−)	LC
18	43/M	B	HCC	14.0	PD	(−)	CH
19	22/M	B	HCC	10.0	PD	(−)	CH
20	67/F	NBNC	HCC	6.0	PD	(−)	Normal
21	52/M	B	HCC	5.0	PD	(+)	LC
22	52/M	B	HCC	5.0	PD	(+)	LC
23	51/M	B	HCC	3.9	PD	(+)	LC
24	26/F	B	HCC/CCC	5.0	PD	(−)	CH
25	56/M	B	HCC	6.0	PD	(−)	LC
26	49/M	B	HCC	3.4	PD	(−)	CH
27	63/M	B	HCC	8.0	PD	(+)	LC

Abbreviations: B, hepatitis B virus; CH, chronic hepatitis; HCC/CCC, combined hepatocellular carcinoma and cholangiocarcinoma; LC, liver cirrhosis; MD, moderately differentiated; NBNC, non-B, non-C; PD, poorly differentiated; Peri-T, peri-tumor; Vas. Inv., vascular invasion.

**Table 2:** Patient and tumor characteristics in Japanese patients.

No	Age/Sex	Virus	Tumor diagnosis	Max. dia. (cm)	Grade	Vas. Inv.	Peri-T liver
1	73/F	C	HCC	2.7	WD	(−)	LC
2	59/F	C	HCC	2.7	WD	(−)	LC
3	59/M	C	HCC	2.5	WD	(−)	LC
4	82/M	C	HCC	3.0	WD	(−)	CH
5	72/M	C	HCC	3.3	WD	(−)	LC
6	62/M	C	HCC	2.0	MD	(−)	CH
7	75/M	C	HCC	2.9	MD	(−)	CH
8	64/M	C	HCC	1.8	MD	(−)	CH
9	77/M	C	HCC	2.0	MD	(−)	CH
10	63/M	C	HCC	1.7	MD	(−)	CH
11	72/M	C	HCC	2.0	MD	(−)	CH
12	78/F	C	HCC	3.7	MD	(−)	CH
13	33/M	B	HCC	2.8	MD	(−)	CH
14	56/M	B	HCC	2.0	MD	(+)	LC
15	78/M	C	HCC	3.3	PD	(+)	CH
16	51/M	C	HCC	3.3	PD	(+)	CH
17	70/M	C	HCC	4.2	PD	(+)	LC
18	63/F	C	HCC	4.5	PD	(+)	LC
19	71/M	C	HCC	6.0	PD	(−)	CH
20	60/F	B	HCC	2.0	PD	(+)	CH

Abbreviations: B, hepatitis B virus; C, hepatitis C virus; CH, chronic hepatitis; LC, liver cirrhosis; Max. dia., maximum diameter; MD, moderately differentiated; PD, poorly differentiated; Peri-T, peri-tumor; Vas. Inv., vascular invasion; WD, well differentiated.

Next, we analyzed the correlation between clinicopathological features and expression of these TCF-4 isoforms (Table 3, Table S1). The results suggest that only the degree(s) of tumor differentiation significantly correlated with TCF-4J and K expression levels (Table 3). Indeed, the TCF-4K level was significantly decreased in poorly differentiated (PD) HCC tumors, which was in contrast to high level of TCF-4J found in those same HCC tumors (Fig. 1A and B, Table 3). Such studies suggest that the upregulated expression of TCF-4J may be a common feature of PD HCC. In support of this hypothesis, TCF-4J was highly expressed in both HBV-related (HepG2, Hep3B, Huh7, and FOCUS) and HCV-related (HAK-1A and HAK-1B) cell lines (Fig. 1C). This isoform was also expressed in OUMS-29 and HEK293. In contrast, TCF-4K expression level was very low in PD HCC tumors and cell lines except for HAK-1B and HEK293 (Fig. 1A, B, and C). In contrast, the two pairs of short isoforms (TCF-4A and B, or G and H) did not demonstrate such a correlation between clinicopathological features and their expression of the SxxSS motif (Table S1).

**Table 3:** Relation between clinicopathological factors and TCF-4J and K expression in patients.

	Group	No	TCF-4J		TCF-4K	
			Level	P value	Level	P value
Age (y)	≤56	23	1.00±0.38	0.2764	0.22±0.23	0.1798
	57≤	24	0.88±0.38		0.31±0.21	
Gender	M	35	0.93±0.41	0.8270	0.27±0.22	0.9107
	F	12	0.96±0.27		0.26±0.24	
Virus	HBV	29	0.98±0.40	0.1991	0.23±0.22	0.1331
	HCV	17	0.83±0.34		0.33±0.21	
Tumorsize (mm)	≤30	17	0.83±0.28	0.1446	0.35±0.19	0.0481
	30<	30	1.00±0.42		0.22±0.23	
Vascularinvasion	−	36	0.88±0.35	0.0521	0.28±0.21	0.3975
	+	11	1.13±0.41		0.21±0.26	
Histology	WD, MD	29	0.82±0.35	**0.0083**	0.35±0.18	**0.0001**
	PD	18	1.12±0.36		0.12±0.21	
Liverbackground	Normal, CH	24	0.98±0.40	0.3997	0.26±0.23	0.9985
	LC	23	0.89±0.36		0.26±0.22	

Abbreviations: CH, chronic hepatitis; HBV, hepatitis B virus; HCV, hepatitis C virus; LC, liver cirrhosis; MD, moderately differentiated; PD, poorly differentiated; WD, well differentiated.

Further analysis demonstrated that TCF-4J expression in HCC was significantly inversely correlated with TCF-4K (Fig. 1D, left panel; *p*  = 0.0031, correlation coefficient *r*  = 0.297). The inverse correlation between TCF-4J and K expression levels was more evident in PD HCC (Fig. 1D, right panel; *p*  = 0.0077, *r*  = 0.437). Thus, loss of the SxxSS motif in the long isoforms of TCF-4 due to a splicing event was associated with a PD HCC phenotype.

### Subcellular Localization of TCF-4J and K Isoforms in the HAK-1A HCC Cell Line

**Figure 2:**
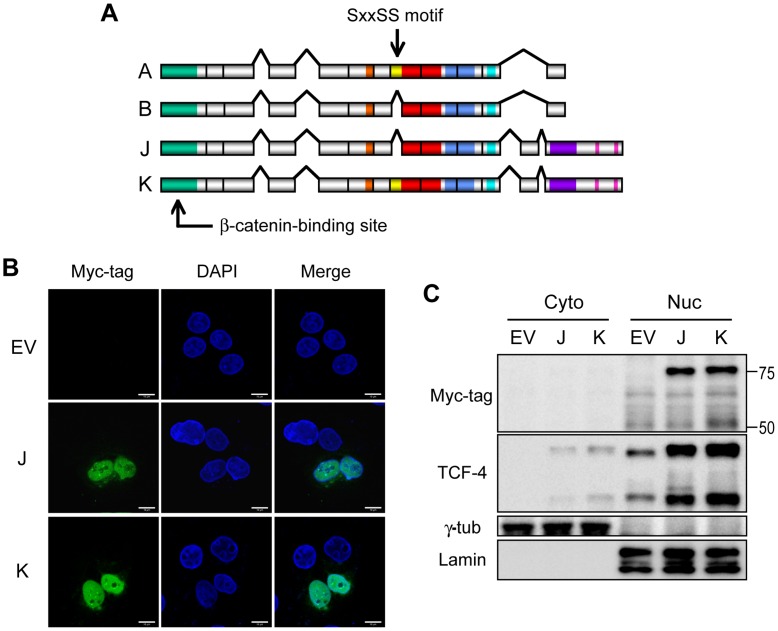
Protein expression of TCF-4 isoforms J and K. (A) Organization of the “short” (A, B) and the “long” (J, K) isoforms; TCF-4B and J lack the SxxSS motif. (B) Confocal laser-scanning microscopy demonstrates nuclear localization of TCF-4J and TCF-4K (green). Nuclei are counterstained by DAPI. (C) Nuclear (Nuc)/cytoplasmic (Cyto) fractionation followed by immunoblot analysis confirms the nuclear localization of TCF-4J and K isoforms. Lamin (lamin A/C) and γ-tubulin were used as nuclear and cytoplasmic markers, respectively.

To better understand how expression of the SxxSS motif may promote the malignant phenotype of HCC *in vitro*, we first examined the localization of TCF-4J and K in HAK-1A HCC cell lines after transient transfection. As shown in Fig. 2, cellular expression was predominantly observed in the nucleus as assessed by confocal microscopy and nuclear/cytoplasmic fractionation studies, suggesting that exogenous expression of TCF-4J and K proteins will localize to the nucleus compartment.

### TCF-4J-overexpressing HCC Cells have a Proliferative Advantage under Hypoxic Conditions

**Figure 3:**
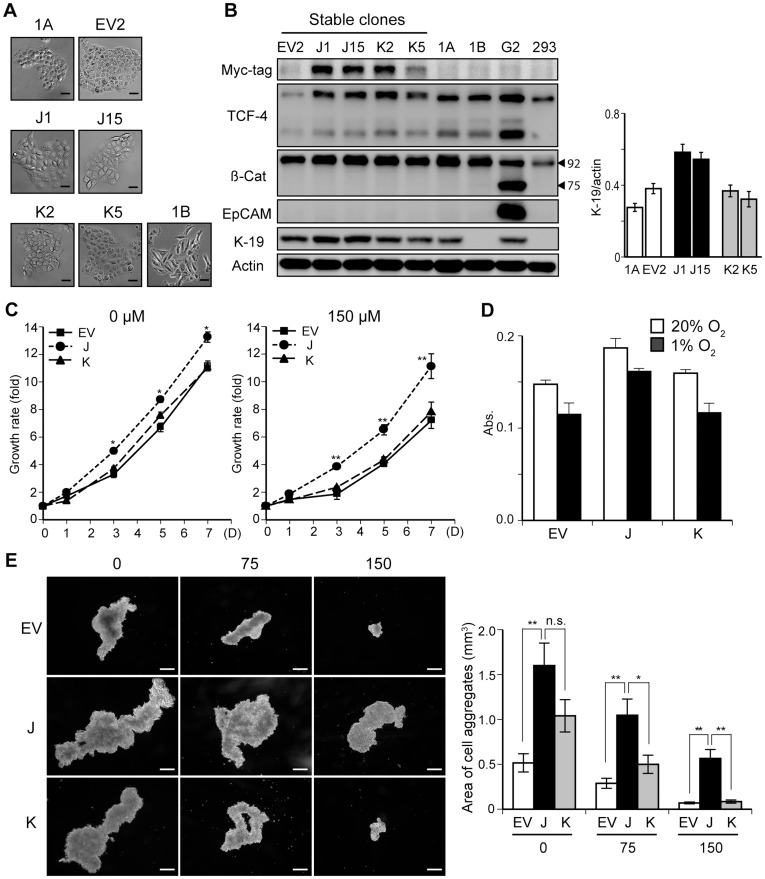
TCF-4J-overexpressing cells proliferate under hypoxic conditions. (A) Phase-contrast microscopy of parental HAK-1A (1A) and 1A-derived stable clones, including the empty vector-transfected clone (EV2), the TCF-4J-transfected clones (J1 and J15), and the TCF-4K-transfected clones (K2 and K5). The morphologic appearance of the highly malignant HAK-1B (1B) cell line, a clonally dedifferentiated cell type from 1A, is also presented for comparison. Bar  = 50 µm. (B) Immunoblot analysis of stable cell clones. Positive signals for Myc-tag are shown in J1, J15, K2, and K5. HepG2 cells (G2) known to express both full-length (92 kDa) and truncated β-catenin (75 kDa), exhibited a lower band for β-catenin (β-Cat) HepG2 was also used as a positive control for EpCAM and K-19 expression. HEK293 was used as a negative control for K-19. (C) Cell growth rates of stable clones under the hypoxic conditions generated by CoCl_2_ (150 µM) for 7 days. Growth rate is represented as the fold-increase compared to day 0. (D) Cell growth of stable clones in normoxia (20% O_2_) or hypoxic conditions (1% O_2_) Cells were exposed to either 20% or 1% oxygen for 5 days. Note that reduction of cell growth in hypoxic contidions was less in J cells (13%) compared to EV (22%) and K (27%) cells. (E) Anchorage-independent growth assay (sphere assay). Phase-contrast microscopic views of representative cell aggregates are displayed at 0, 75, and 150 µM of CoCl_2_. Bar  = 300 µm. Note the striking difference in colony growth rate and appearance at 150 µM of CoCl_2_. *p<0.05; **p<0.01.

During the multi-step process of hepatocarcinogenesis, less differentiated HCC cells arise from the center of HCC tumor nodules revealing a “nodule-in-nodule” histologic appearance on pathologic examination of tissue sections [28]. The central tumor area generates hypoxic stress to tumor cells and promotes apoptosis [18], [29]. However, there may be a hypoxia-resistant population of HCC cells that survive under these severe conditions to promote tumor growth through accelerated angiogenesis and vascular invasion. These two phenomena are regulated by HIF-1α and HIF-2α [30], [31]. Therefore, we hypothesized that the high expression of TCF-4J in PD HCC was a possible reflection of an induced hypoxia-resistant HCC phenotype. To test this idea, stable cell clones overexpressing TCF-4J (J cells) and TCF-4K (K cells) were established, and comparisons were made to empty vector-transfected cell clones (EV cells), as well as to the parental HAK-1A cells. Under phase-contrast microscopy, the morphologic appearance of these clones was similar and comparable to the parental cells (Fig. 3A), which was in contrast to the HAK-1A-derived dedifferentiated cell clone HAK-1B [24].

Wnt/β-catenin signaling often correlates with stem cell-like molecular alterations [32], [33]. In this regard, the expression of EpCAM, a putative liver cancer stem cell (CSC) marker and a β-catenin/TCF-4 downstream target gene product [6], was not upregulated in these cells. However, the expression of K-19, a biliary lineage marker for an aggressive HCC phenotype [34], was increased in J cells but not in K, EV, or parental HAK-1A cells (Fig. 3B).

Consistent with our previous report in Huh7 cells [13], J cells had an increased basal growth rate compared to K and EV control cells. The J cells were resistant under chemically induced, severe hypoxic conditions (150 µM CoCl_2_), proliferated significantly faster than K or EV cells (Fig. 3C). This finding was further supported when cells were exposed to 1% oxygen for 5 days (Fig. 3D). Cell growth in hypoxia (1% oxygen) was reduced in EV and K cells compared to cell proliferation in normoxia (20% oxygen) by 22 and 27%, respectively, while J cells demostrated only a 13% reduction in cell growth. Moreover, robust resistance to severe hypoxia in the J cells was also exhibited in a sphere assay. In this context, the J cells formed the largest cell aggregates at all the CoCl_2_ concentrations employed. The J cells’ potential for anchorage-independent growth was most evident with severe hypoxia induced by 150 µM CoCl_2_ (Fig. 3E).

### Loss of the SxxSS Motif Contributes to the Expression Levels of HIF-αs

**Figure 4:**
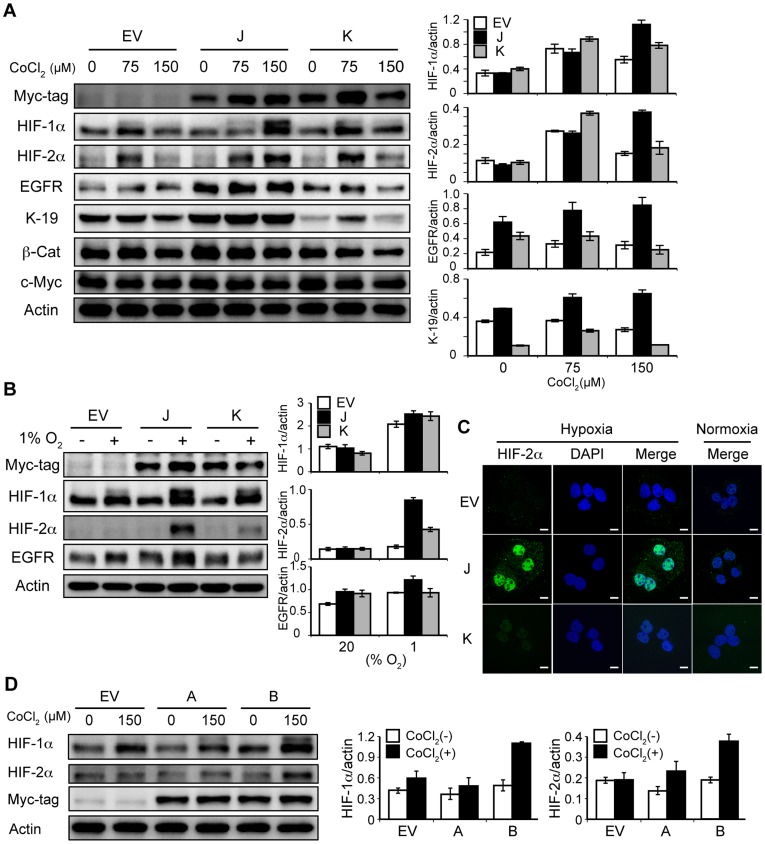
Loss of the SxxSS motif in TCF-4 isoforms increases expression of HIF-αs under hypoxia. (A and B) Immunoblot analysis of empty vector-transfected (EV), TCF-4J-overexpressing (J), and TCF-4K-overexpressing cells (K). The cells were treated with 0 and 150 µM CoCl_2_ (A) or cultured under 1% oxygen tension for 48 hr (B). Cell lysates were subjected to detect HIF-αs and Myc-tag TCF-4 isoform protein expression. Expression levels were plotted as a ratio to actin (right panel). (C) Confocal microscopy for nuclear localization of HIF-2α protein. Cells were treated with 0 µM (Normoxia) or 150 µM (Hypoxia) CoCl_2_ and stained with an anti-HIF-2α antibody (green). DAPI was employed for nuclear staining. (D) Immunoblot analysis of total cell lysates from cells treated with 0 or 150 µM CoCl_2_ for 48 hr. A and B represent HAK-1A cells overexpressing TCF-4A (“short form” of TCF-4K) and TCF-4B (“short form” of TCF-4J), respectively (see Figure 2A). Note the robust increase in expressions of HIF-1α and HIF-2α in B cells.

Based on the hypoxia-resistant features exhibited by J cells, we determined if these cells expressed HIF-αs under hypoxia. Both HIF-1α and HIF-2α expression in J and K cells were explored since HIF-2α has recently been shown to be a key molecule that promotes survival of CSCs under hypoxic conditions [35], [36], [37]. As expected, HIF-1α and HIF-2α expression were induced with moderate hypoxia (75 µM CoCl_2_) in EV, J and K cells; however, under severe hypoxic conditions (150 µM CoCl_2_), only J cells maintained these high protein levels (Fig. 4A). Stabilized HIF-2α, often found in the hypoxic core of the tumor, upregulated the expression of EGFR which may contribute to tumor growth [18], and the activation of EGFR signaling pathway was associated with the development of K-19-positive HCC [38]. In J cells, expression of EGFR and K-19 was associated with an increased level of HIF-2α (Fig. 4A). The consistent HIF response was also observed in 1% oxygen-exposed J cells (Fig. 4B). Furthermore, the 150 µM CoCl_2_-induced HIF-2α expression was localized in the nuclei of J cells as assessed by immunofluorescence staining (Fig. 4C).

We also determined if HIF-αs upregulation under severe hypoxia in J cells was linked to loss of the SxxSS motif. If that were the case, a similar result would be found in the TCF-4B overexpressing cells (B cells), the so-called “short isoform” of the TCF-4J (Fig. 2A). In this regard, total cell lysates were subjected to immunoblot analysis to compare B with A cells where TCF-4A is overexpressed as the “short form” counterpart of TCF-4K (Fig. 2A). It is important to note that an increase in total cell lysate HIF-α expression was evident in B cells (Fig. 4D) and suggests that the SxxSS motif may have a regulatory role in modulating HIF-αs expression under severe hypoxic conditions generated by 150 µM CoCl_2_ in two pairs of TCF-4 isoforms.

### Accelerated Ubiquitin-dependent Degradation of HIF-αs in K Cells

**Figure 5:**
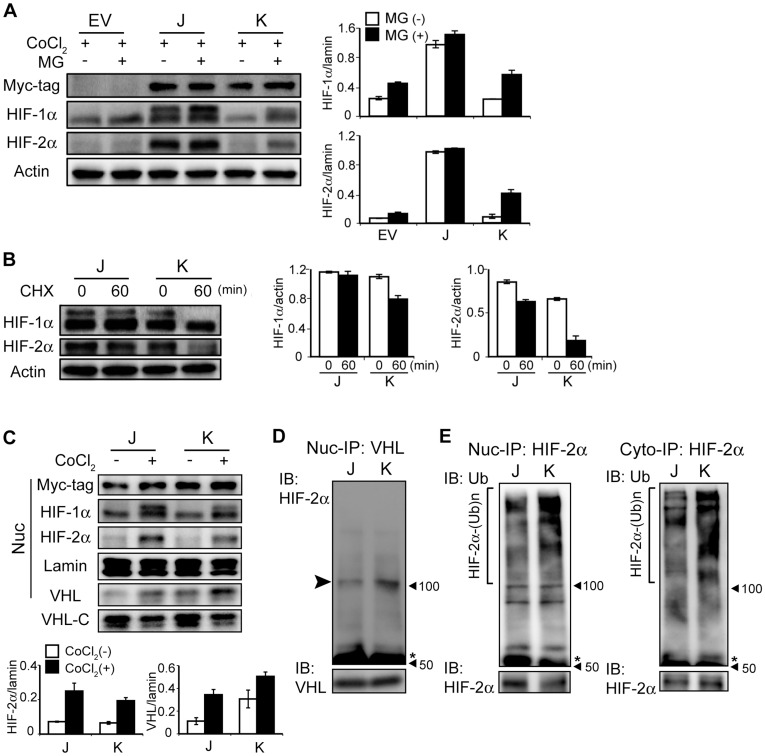
Lack of SxxSS motif contributes to protein stability of HIF-αs in J cells. (A) Cells were treated with 150 µM CoCl_2_ for 36 hr followed by incubation with or without MG-132 (10 µM) for 2 hr. Total cell lysates were employed for immunoblot analysis. Relative expression of HIF-αs was normalized to actin. (B) Protein stability of HIF-αs was evaluated by immunoblot analysis, where cells were exposed to 5 µg/mL cycloheximide (CHX) to inhibit protein synthesis for 0 or 60 min at the last phase of the 48 hr CoCl_2_ treatment period. (C) Expression of VHL in TCF-4J and K cells. Cells were treated 0 or 150 µM CoCl_2_ and nuclear and cytoplamic fractions was prepared for immunoblot analysis. Expression level of HIF-2α and VHL was normalized by lamin (bottom panel); VHL-C, cytoplasmic VHL. (D) Interaction between VHL and HIF-2α in nucleus of TCF-4J and K cells. (E) Polyubiquitination of HIF-2α in cytoplasmic and nuclear fractions of TCF-4J and K cells. Immunoprecipitation (IP)/immunoblot (IB) analysis was performed by using antibodies for HIF-2α and ubiquitin (Ub); *, IgG heavy chain. Note the decreased ubiquitination of HIF-2α and weak VHL-HIF-2α interaction in J cells, which was in contrast to the observations in K cells.

Because HIF-αs proteins are highly degraded by the ubiquitin-dependent proteasomal pathway, it was hypothesized that a differential HIF-αs degradation mechanism may exist under severe hypoxia in J and K cells. The levels of HIF-1α and HIF-2α were substantially increased in K cells treated with a proteasomal inhibitor (MG-132) compared to non-treated K cells; in contrast we observed less of an increase in these proteins in EV and J cells (Fig. 5A). Accumulation of HIF-αs in the MG-132-treated K cells strongly suggests that SxxSS motif-harboring K cells may degrade HIF-αs under hypoxic conditions in a proteasomal-dependent manner. Indeed, protein stability of HIF-αs was found to be decreased in K cells (Fig. 5B). We found that the level of VHL protein, a known E3 ligase for HIF-αs, in K cells was higher compared to J cells in the nucleus, but not cytoplasm, which coincided with intense nuclear accumulation of HIF-1α and HIF-2α in J cells under hypoxic conditions (Fig. 5C). We further examined interaction between HIF-2α and VHL on nuclear extracts and found that nuclear VHL in K cells strongly interacted with HIF-2α compared to J cells to promote the HIF-2α ubiquitination in the nucleus (Fig. 5D). Moreover, K cells revealed poly-ubiquitination of HIF-2α compared to J cells which supports the concept of accelerated degradation of HIF-α in K cells (Fig. 5E). This finding implies a possible role for nuclear VHL in regulating stability of HIF-αs in the TCF-4 isoform-overexpressing cells.

### TCF-4J Isoform Expression Confers a Tumorigenic Phenotype to HCC Cells

**Figure 6:**
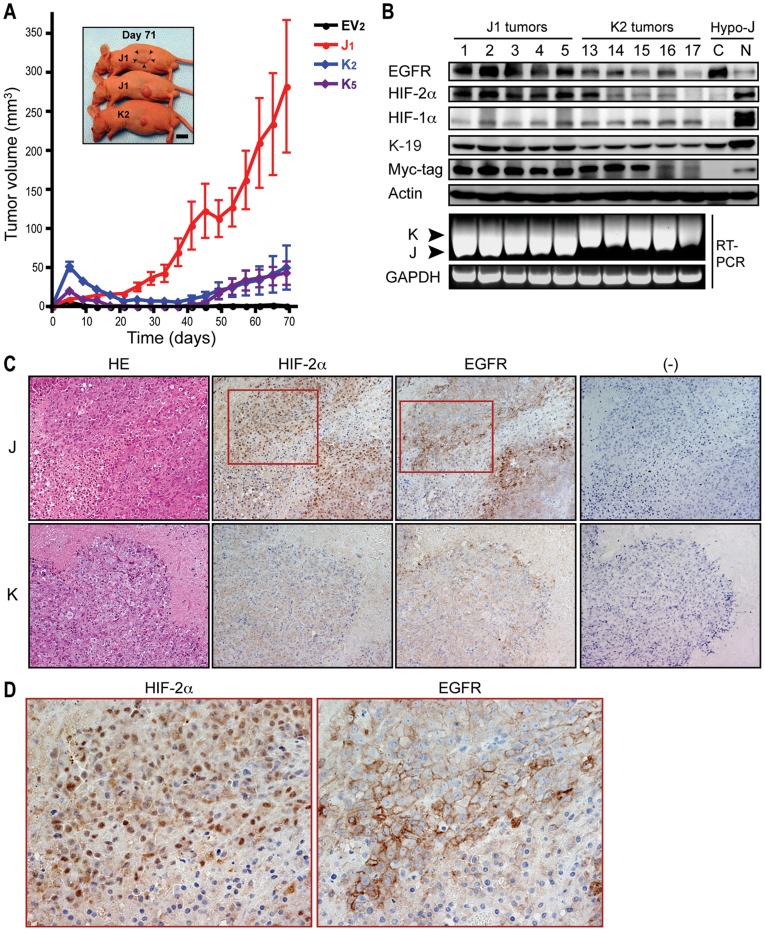
Loss of the SxxSS motif in TCF-4 isoforms promotes tumorigenesis. (A) Representative experiment demonstrating xenograft tumor development and growth rate. EV2, control; J1, J cell; K2 and K5, K cell clones. (B) Protein expression of the indicated molecules in J1 tumors (1–5) and K2 tumors (13–17) by immunoblot analysis. Nuclear (N) and cytoplasmic (C) proteins expressed in the 150 µM CoCl_2_-treated J cells (Hypo-J) were used as positive controls. (Bottom panel) TCF-4J and K mRNA expression was verified by RT-PCR in J1 and K2 tumors; GAPDH, glyceraldehyde-3-phosphate dehydrogenase. (C) Immunohistochemistry for HIF-2α and EGFR expression in representative J- and K-cell derived tumors. Original magnification, 200x. HE, hematoxylin and eosin; and (-), negative staining. (D) Magnified view (400x) for the corresponding squared areas in (C) for HIF-2α and EGFR expression.

The tumorigenic potential of EV, J, and K cells was assessed in nude mice. As might be predicted from the *in vitro* findings, J cells were highly tumorigenic. Although K cells generated small tumors, they appeared later (about 40 days) after tumor cell injection and grew very slowly (Fig. 6A). Control (EV) cells did not produce tumors as reported previously [24].

Immunoblot analysis was applied to confirm whether the xenograft tumors had the same protein phenotype as exhibited in the cell clones. The Myc-tag detected exogenous TCF-4J and K isoforms derived from tumor tissues; in addition, RT-PCR was performed to verify overexpression of the TCF-4J or K without cross-tissue contamination during these experiments (Fig. 6B; lower panel). Consistent with previous report that HIF-2α upregulated EGFR expression in the hypoxic core of solid tumors [18], higher expression levels of EGFR in concert with HIF-2α were found in J compared to K cell-derived tumors (Fig. 6B).

**Figure 7:**
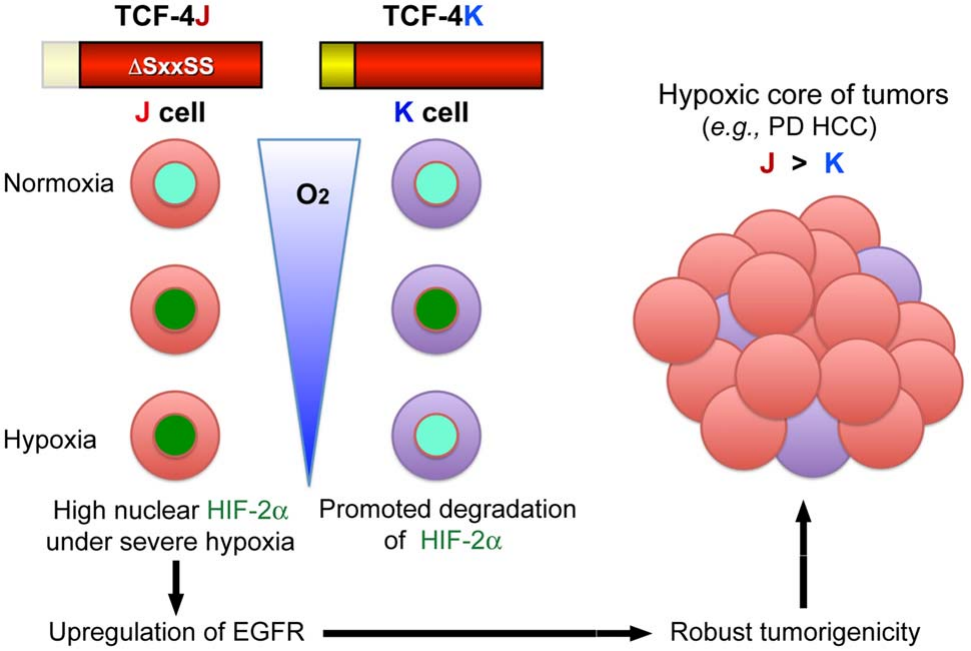
Proposed role(s) of TCF-4J and K isoforms during hepatic oncogenesis. Loss of SxxSS motif in TCF-4J (ΔSxxSS) supports high level nuclear expression of HIF-2α with increasing degrees of hypoxia. This phenomenon upregulates and activates the EGFR signaling cascade including downstream genes that inhibit apoptosis to promote tumor development. In contrast, the TCF-4K isoform containing the SxxS motif degrades HIF-2α under increasing hypoxic conditions via the ubiquitination pathway and, therefore, downregulates HIF-2α mediated gene expression. The net effect is to inhibit the characteristics of a more aggressive malignant phenotype. Thus, TCF-4 isoform expression may play an important role in the molecular pathogenesis of poorly differentiated HCC where severe hypoxia is likely to occur and particularly when TCF-4 isoforms that lack a regulatory SxxSS motif are expressed.

We also assessed HIF-2α and EGFR protein expression in xenograft tumors by immunohistochemistry. The histological features of the tumor tissue as well as cellular morphology did not differ among tumors induced by either J or K cells using hematoxylin and eosin staining. However, there was increased expression of both HIF-2α and EGFR predominantly in the J cell-derived tumor tissue (Fig. 6C). Since tumors frequently contain central necrotic areas that form a boundary between the areas of living and hypoxic dead tumor cells [29], we focused on the immunoreactive signals at this interface. Striking nuclear and cytoplasmic expression of HIF-2α and membranous EGFR were predominantly found at the hypoxic boundary in J cell derived tumor tissues (Fig. 6C and D).

## Discussion

In the present study, we demonstrated the following: (1) in hepatocyte derived cells, overexpression of the TCF-4J isoform lacking the SxxSS motif, exhibited robust tumorigenic potential; (2) the TCF-4J isoform was highly expressed in rapidly growing and presumably in the hypoxic milieu of human PD HCC, and (3) TCF-4J expression contributed to nuclear accumulation of HIF-2α and was due to, in part, less ubiquitin-dependent degradation of HIF-αs in J cells compared to K cells under severe hypoxia. This observation was linked to reduced expression of VHL.

There is evidence that genetic and/or epigenetic deregulation of the Wnt/β-catenin signaling pathway leads to tumor formation [2]. Indeed, genetic mutations in components of this pathway such as *APC*, *AXIN1/2* and *CTNNB1* are well-established molecular events in colorectal, as well as gastric carcinomas and HCC [39], [40]. Epigenetic disruptions in the Wnt/β-catenin signaling are also involved in oncogenesis through inappropriate stabilization of β-catenin and aberrant activation of upstream components of this signal transduction cascade [9], [10], [41]. There is little information, however, on the potential role of TCF-4 transcription factor isoforms in the oncogenic process. We demonstrated that overexpression of human TCF-4 isoforms may promote hepatic oncogenesis through actions of their intrinsic motifs which suggest that the Wnt/β-catenin signaling cascade may be augmented and further able to contribute to the malignant phenotype due to mechanisms that are operative in the nucleus. Indeed, expression analysis in human HCC tumors revealed that both TCF-4J and TCF-4K were present in non-cancerous dysplastic and normal liver, although both isoforms were initially identified and cloned from HCC cell lines. This finding is consistent with the previous report that emphasized the physiological distribution of TCF-4 isoforms in mice [42]. In human HCC tissues, however, PD HCCs demonstrated reduced expression of TCF-4K and exhibited very high levels of TCF-4J. The differential expression pattern of this TCF-4 isoform pair was independent of viral etiology and more directly related to the hypoxic microenvironment characteristic of PD HCC.

Although the precise interactions between HIF-αs and the β-catenin/TCF-4 complex are under evaluation, it has been demonstrated that HIF-1α [16] and HIF-2α [17] directly interact with β-catenin and thereby modulate TCF-4-mediated transcriptional activity. These observations suggest an important role for tissue hypoxia in regulating the Wnt/β-catenin/TCF-4 signaling cascade as a mechanism, which contributes to hepatic oncogenesis. Furthermore, the role of the SxxSS motif in mediating TCF-4 effects on HIF-αs stability under hypoxic conditions has been emphasized here. We found that TCF-4J and TCF-4B, which lack this motif, are associated with enhanced expression of HIF-1α and HIF-2α under severe hypoxia, whereas TCF-4K and TCF-4A, which express the SxxSS sequence, do not exhibit this property. As a potential mechanism(s), we found less ubiquitin-dependent degradation of HIF-αs in J than K cells under severe hypoxia; less degradation in J cells was linked to reduced expression of VHL. In contrast, the HIF-αs were highly degraded in K cells, raising the possibility that HIF-αs were subjected to VHL-mediated degradation due to the presence of the SxxSS motif. These findings emphasize the possible involvement of TCF-4 isoforms in mediating HIF-α stability via this SxxSS motif; however, it is unclear whether each S of the SxxSS plays a distinct role in the regulation of HIF-α stability.

The biological roles of HIF-1α and HIF-2α under hypoxia is controversial; however, HIF-2α, but not HIF-1α, is considered to be important in tumor formation [43], by promoting Myc activity [44]. In addition, HIF-2α is known to upregulate EGFR expression in the hypoxic core of solid tumors [18]. Emerging evidence has been presented that HIF-2α should be recognized as a CSC marker [45]. It is of interest that HIF-2α expression was significantly associated with poor prognosis in aggressive gliomas [37]. Since TCF-4J expression in our study was a potential biomarker for an aggressive HCC phenotype [34], we focused on the possible relevance of SxxSS as a mechanism of HIF-2α stabilization under hypoxic conditions. Since K cells showed weak and little tumorigenic ability in xenograft model, it was suggested that mechanism of drastic HIF-2α degradation in those cells even under hypoxia worked also may occur *in vivo*, possibly through affecting the EGFR expression in a SxxSS-dependent manner. It was observed in xenograft tissues that the driving force (HIF-2α) for EGFR expression was active in J cells as compared to K cells, especially in tumor cells under hypoxic conditions as observed in necrotic area. This HIF-2α-induced upregulation of EGFR in J cells may be associated with activation of EGFR signaling pathways and further studies will be needed.

The robust tumorigenic potential of J cells may be predicted by their significant anchorage-independent growth ability *in vitro* under hypoxic conditions. The oncogenic potential of J cells grown as xenografts is likely to be triggered by chronic hypoxic stress derived from subcutaneous inoculation which has previously been shown to have a poor neovascularization and thus, would generate a hypoxic environment during rapid tumor growth. The expression levels of both HIF-2α and EGFR were much higher in J tumors than K derived tumors and suggests that the J tumors may have grown faster through activating the HIF-2α/EGFR system under a hypoxic environment as summarized by the illustration presented in Fig. 7. This hypoxia-resistant property of J cells may be the biologic basis for the finding that TCF-4J was the predominant isoform expressed in human PD HCCs, which are known to have a hypoxic core in the central region of the tumor [18], [29].

## Supporting Information

Figure S1 **Expression levels of TCF-4A, B, G and H isoforms in human HCCs. (**A and C) Comparative analysis of TCF-4A/B (A) and G/H (C) mRNA expression levels in 27 HBV-related HCC tumors (T) adjacent peritumor tissue (pT) and histologic normal liver (N) by RT-PCR. (B and D) Comparative analysis of TCF-4A/B (B) and G/H (D) mRNA expression levels in another 20 HCC tumors including five WD HCCs from a different clinical site. Seventeen individuals had HCV-related chronic liver disease, and the remainder had chronic HBV infection. Values are normalized to GAPDH. Statistical results from all tissues are expressed as mean + SD (right panel). MD, moderately differentiated; PD, poorly differentiated; WD, well differentiated; *p<0.01; **p<0.05
(TIF)

Table S1 **Relation between clinicopathological factors and TCF-4A and B, and G and H expression in patients.**
(DOC)
