# Supplementary figures and images for: Loss of the SxxSS Motif in a Human T-Cell Factor-4 Isoform Confers Hypoxia Resistance to Liver Cancer: An Oncogenic Switch in Wnt Signaling
Source: PLoS One. 2012 Jun 29;7(6):e39981. doi: 10.1371/journal.pone.0039981 (PMC3386968; doi:10.1371/journal.pone.0039981)

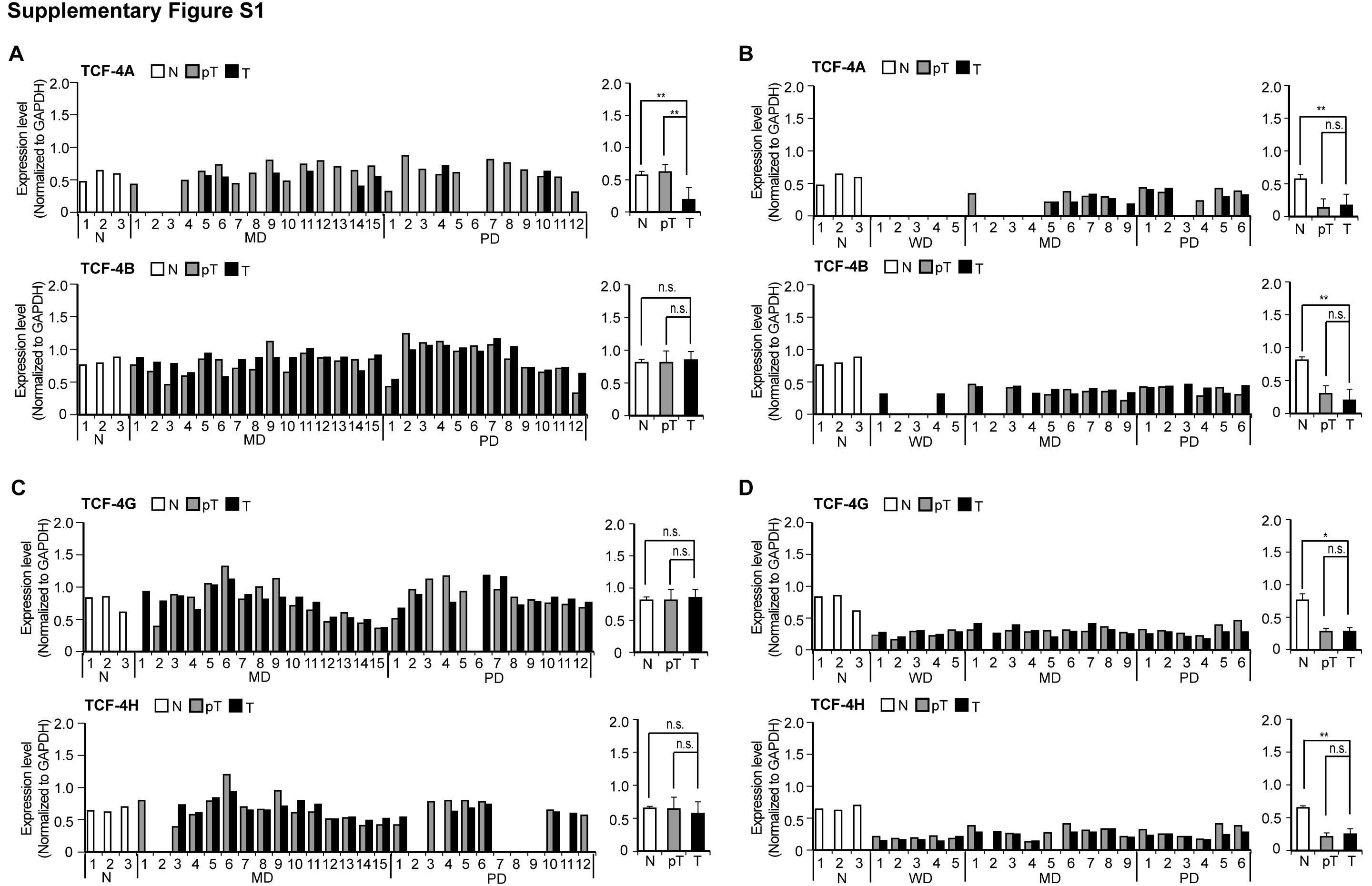

Supplement: Figure S1 — Expression levels of TCF-4A, B, G and H isoforms in human HCCs. (A and C) Comparative analysis of TCF-4A/B (A) and G/H (C) mRNA expression levels in 27 HBV-related HCC tumors (T) adjacent peritumor tissue (pT) and histologic normal liver (N) by RT-PCR. (B and D) Comparative analysis of TCF-4A/B (B) and G/H (D) mRNA expression levels in another 20 HCC tumors including five WD HCCs from a different clinical site. Seventeen individuals had HCV-related chronic liver disease, and the remainder had chronic HBV infection. Values are normalized to GAPDH. Statistical results from all tissues are expressed as mean + SD (right panel). MD, moderately differentiated; PD, poorly differentiated; WD, well differentiated; *p<0.01; **p<0.05 (TIF) [file pone.0039981.s001.tif]
