# Supplementary material for: Loss of the SxxSS Motif in a Human T-Cell Factor-4 Isoform Confers Hypoxia Resistance to Liver Cancer: An Oncogenic Switch in Wnt Signaling
Source: PLoS One. 2012 Jun 29;7(6):e39981. doi: 10.1371/journal.pone.0039981 (PMC3386968; doi:10.1371/journal.pone.0039981)
Supplement: Table S1 — Relation between clinicopathological factors and TCF-4A and B, and G and H expression in patients. (DOC) [file pone.0039981.s002.doc]

**Supple Table S1.** Relation between clinicopathological factors and TCF-4A and B, and G and H expression in patients.

|  | **Group** | **No** | **TCF-4A** | |  | **TCF-4B** | |  | **TCF-4G** | |  | **TCF-4H** | |
| --- | --- | --- | --- | --- | --- | --- | --- | --- | --- | --- | --- | --- | --- |
|  |  |  | **Level** | **P value** |  | **Level** | **P value** |  | **Level** | **P value** |  | **Level** | **P value** |
| Age (y) | ≤ 56 | 23 | 0.24±0.32 | 0.7485 |  | 0.87±0.24 | **0.0151** |  | 0.76±0.24 | 0.0685 |  | 0.45±0.32 | 0.5779 |
|  | 57 ≤ | 24 | 0.21±0.31 |  |  | 0.68±0.33 |  |  | 0.63±0.23 |  |  | 0.49±0.22 |  |
| Gender | M | 35 | 0.21±0.31 | 0.6189 |  | 0.77±0.28 | 0.9765 |  | 0.72±0.22 | 0.2001 |  | 0.45±0.28 | 0.4274 |
|  | F | 12 | 0.26±0.33 |  |  | 0.77±0.27 |  |  | 0.61±0.29 |  |  | 0.53±0.23 |  |
| Virus | HBV | 29 | 0.22±0.29 | 0.8166 |  | 0.85±0.16 | 0.0980 |  | 0.76±0.24 | **0.0292** |  | 0.48±0.32 | 0.6801 |
|  | HCV | 17 | 0.24±0.35 |  |  | 0.63±0.38 |  |  | 0.61±0.16 |  |  | 0.45±0.17 |  |
| Tumor size (mm) | ≤ 30 | 17 | 0.19±0.27 | 0.5632 |  | 0.66±0.33 | **0.0320** |  | 0.69±0.16 | 0.9383 |  | 0.49±0.24 | 0.7773 |
|  | 30 < | 30 | 0.24±0.33 |  |  | 0.84±0.22 |  |  | 0.69±0.28 |  |  | 0.46±0.29 |  |
| Vascular invasion | - | 36 | 0.21±0.29 | 0.5999 |  | 0.73±0.29 | **0.0437** |  | 0.68±0.24 | 0.5803 |  | 0.48±0.26 | 0.5832 |
|  | + | 11 | 0.27±0.39 |  |  | 0.92±0.15 |  |  | 0.73±0.26 |  |  | 0.43±0.31 |  |
| Histology | WD, MD | 29 | 0.20±0.27 | 0.6257 |  | 0.70±0.30 | **0.0196** |  | 0.71±0.19 | 0.4396 |  | 0.53±0.24 | 0.0627 |
|  | PD | 18 | 0.25±0.37 |  |  | 0.89±0.18 |  |  | 0.65±0.31 |  |  | 0.38±0.29 |  |
| Liver background | Normal, CH | 24 | 0.28±0.35 | 0.2189 |  | 0.80±0.22 | 0.4786 |  | 0.65±0.25 | 0.2888 |  | 0.50±0.26 | 0.4273 |
|  | LC | 23 | 0.16±0.26 |  |  | 0.74±0.33 |  |  | 0.73±0.23 |  |  | 0.44±0.28 |  |

Abbreviations: CH, chronic hepatitis; HBV, hepatitis B virus; HCV, hepatitis C virus; LC, liver cirrhosis; MD, moderately differentiated; PD, poorly differentiated; WD, well differentiated
